# Editorial: Medicine and Society

**DOI:** 10.3389/fmed.2020.570551

**Published:** 2020-10-14

**Authors:** Pietro Ghezzi, Arianne Shahvisi, Hilde Stevens

**Affiliations:** ^1^Brighton and Sussex Medical School, Brighton, United Kingdom; ^2^Institute for Interdisciplinary Innovation in Healthcare, Université libre de Bruxelles, Brussels, Belgium

**Keywords:** pseudoscience, ethics, health information, genetic testing, patient involvement

This Research Topic on Medicine and Society focusses on different aspects of health information and their impact on the concept of personalized medicine. Members of the public are increasingly able to access large amounts of information, which raises important issues about quality, ideology, and commercial bias in the material available on the Internet. Rather than empowering patients and communities, which is a key aim for personalized medicine, biased or false information may promote the unregulated sale of health-related products and services online for which there is no evidence of efficacy. The degree to which individuals are capable of obtaining, processing, and understanding health information to make informed decisions related to preventing, curing, or living with diseases—briefly, their health literacy—greatly impacts their quality of life. Patient-centricity and patient empowerment is a growing movement that supports patients, and the wider public, in developing the skills needed to process health information and engage as an active partner in the drug development life cycle. Improving the quality of healthcare starts with improving the quality of health information made available to the public.

While many studies have proposed frameworks that define the dimensions of information quality, these are not easily applicable to health information. This issue's study by Al-Jefri et al., based on a survey, attempts to define the dimensions and categories of health information quality, in particular, examining the perspective of consumers.

The contribution by Cassa Macedo et al. analyzes online information relating to growing demands to “boost the immune system” with a variety of supplements and highlights the commercial interests driving this concept.

Another field where the commodification of science is generating new information with ambiguous meanings is that of genetic testing. Direct-to-consumer genetic testing is a growing market. While the focus was initially on risk factors, such as BRCA mutations as risk factors for breast cancer, which have a firm scientific basis, the emphasis is shifting to questions of identity, where the science is questionable and has grave social implications. Blell and Hunter explore the more recent development of DNA tests aimed at determining “genetic ancestry,” which often misrepresent and oversell the scope of genetics, and may be used to strengthen harmful myths of biological racial categories, which frustrates efforts toward justice and personalization in healthcare.

While the focus in medicine is often on medical professionals and pharmaceutical companies, the evolving landscape, represented by online resources and the commodification of science, emphasizes the need to ensure that knowledge production is more democratic and discursive. To this end, Grine et al. report on the activity of EUPATI Belgium, emphasizing the importance of patient involvement in the research and development of medicinal products. The study highlights the challenges of ensuring that accurate information is available and accessible, and stresses the importance of open infrastructures which are easily available to all those who need them.

## Author Contributions

All authors listed have made a substantial, direct and intellectual contribution to the work, and approved it for publication.

## Conflict of Interest

The authors declare that the research was conducted in the absence of any commercial or financial relationships that could be construed as a potential conflict of interest.

